# Supplementary figures and images for: Use of the Health Improvement Card by Chinese physical therapy students: A pilot study
Source: PLoS One. 2019 Sep 5;14(9):e0221630. doi: 10.1371/journal.pone.0221630 (PMC6728073; doi:10.1371/journal.pone.0221630)

S1 Appendix English version of the Health Improvement Card


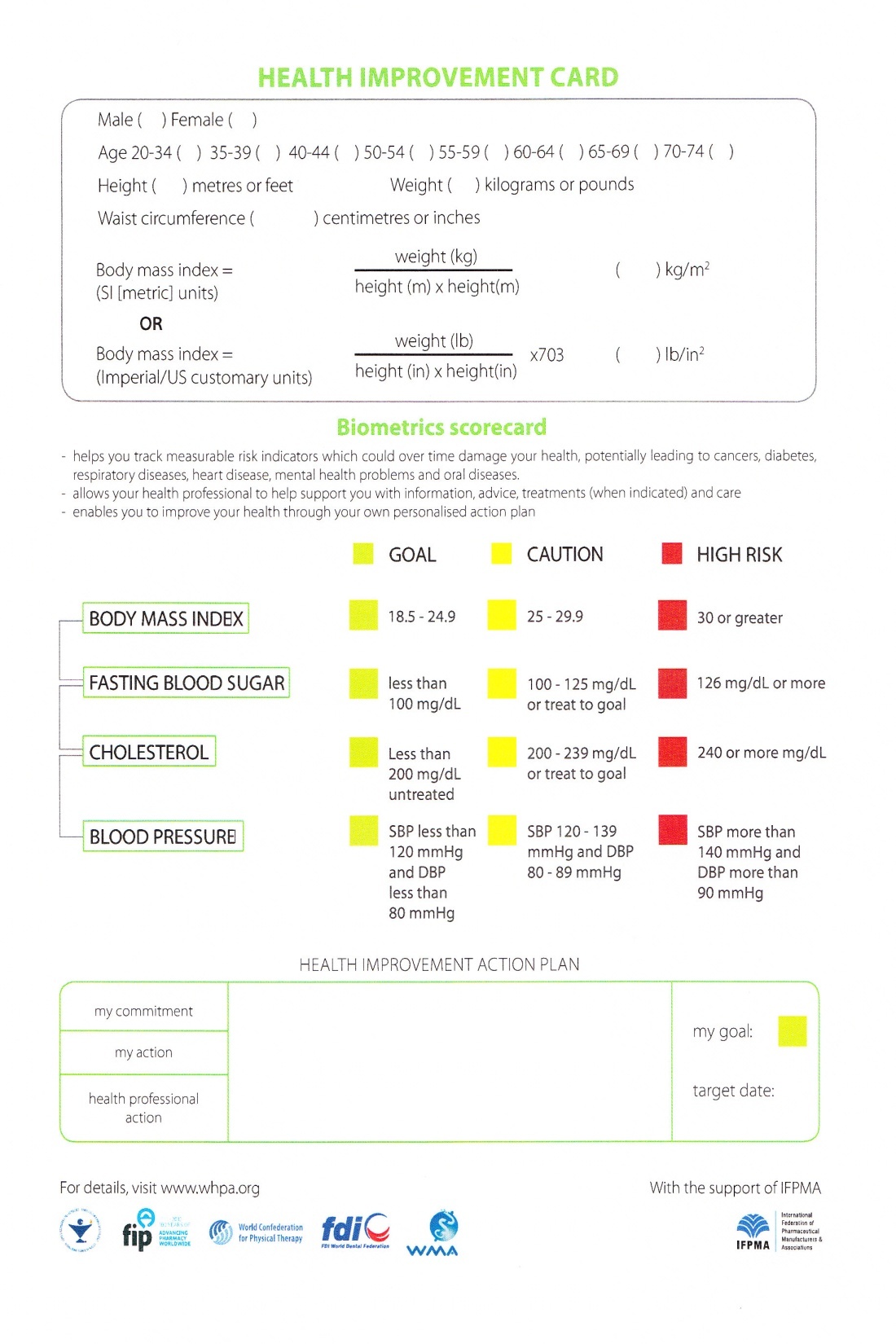


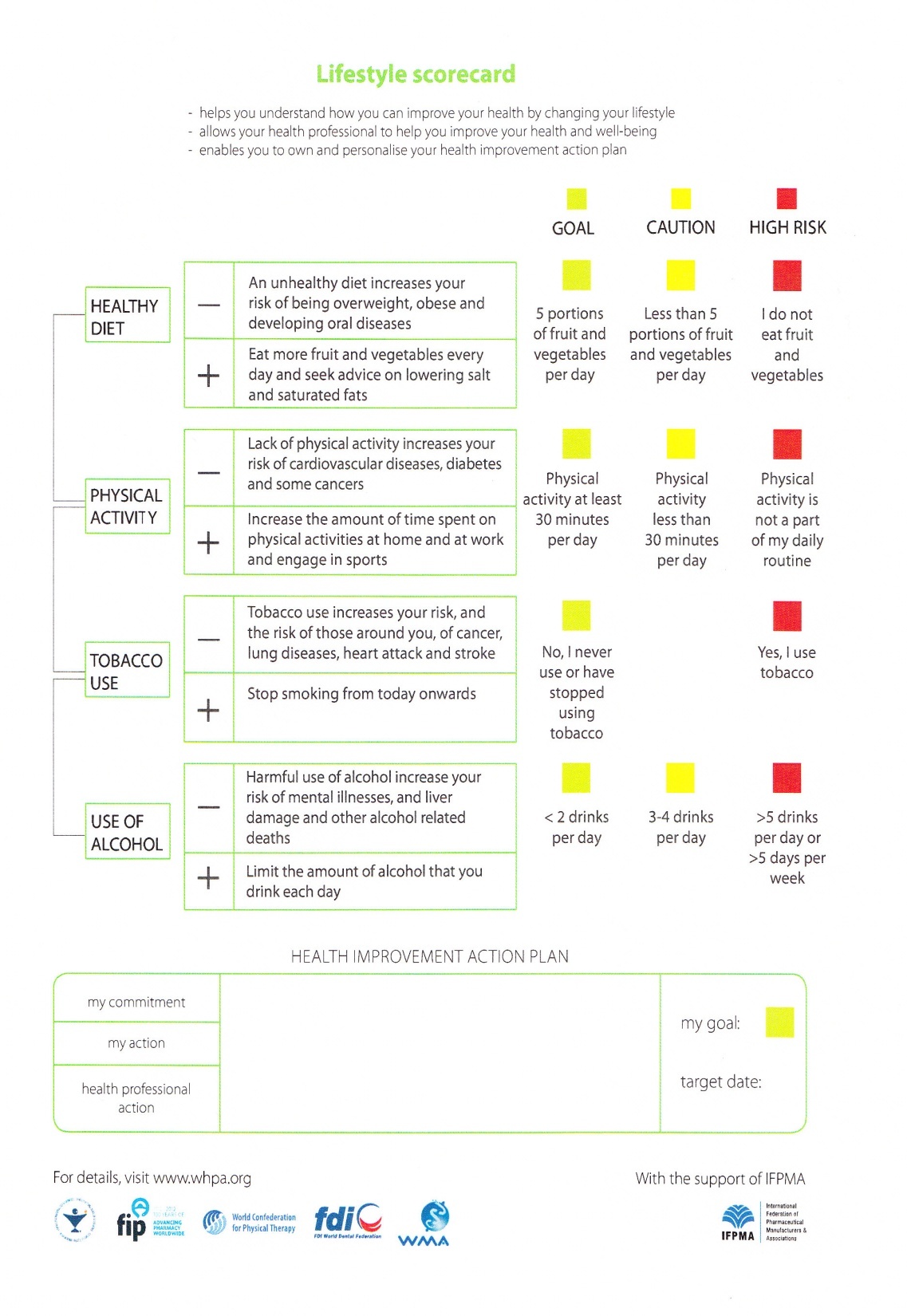

Supplement: S1 Appendix — (DOCX) [file pone.0221630.s001.docx]

S2 Appendix Chinese version of the Health Improvement Card


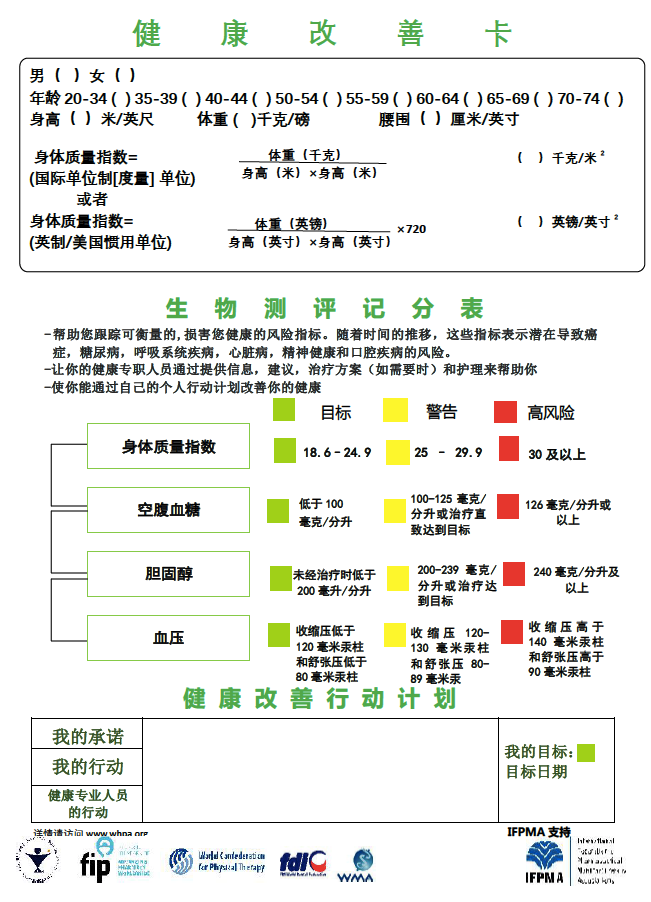

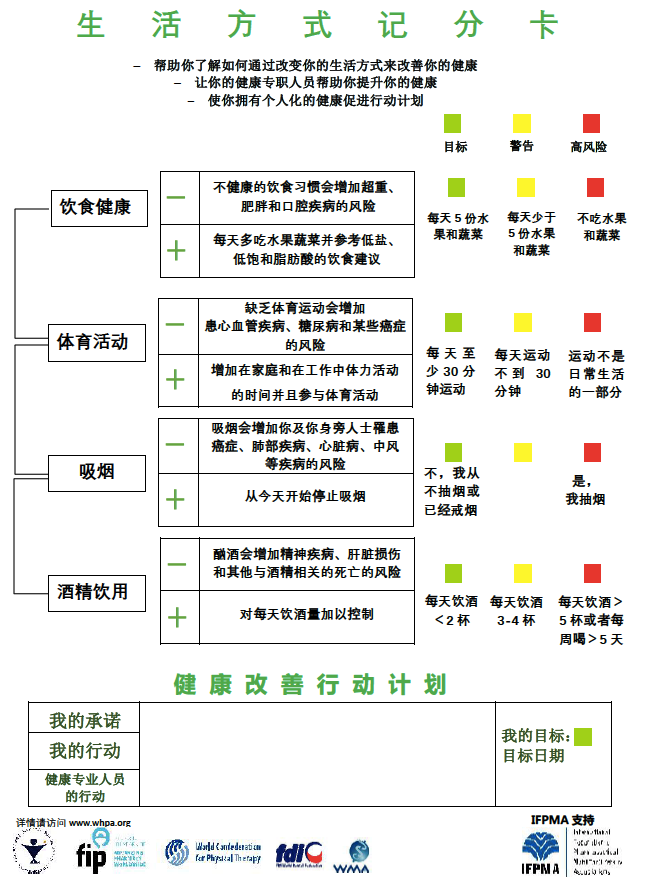

Supplement: S2 Appendix — (DOCX) [file pone.0221630.s002.docx]
