# Supplementary material for: Use of the Health Improvement Card by Chinese physical therapy students: A pilot study
Source: PLoS One. 2019 Sep 5;14(9):e0221630. doi: 10.1371/journal.pone.0221630 (PMC6728073; doi:10.1371/journal.pone.0221630)
Supplement: S3 Appendix — (DOCX) [file pone.0221630.s003.docx]

S3 Appendix Students’ perceptions of the use and application of the Health Improvement Card

Please answer the following questions as honestly as possible.

SA=strongly agree, A=agree, DA=disagree, SD=strongly disagree

SA A DA SD

1. Physical therapists should introduce the Health Improvement Card to the general public
2. I understand the purpose and role of the Health Improvement Card
3. I can provide advice to my patients about the actions prescribed on the Health Improvement Card
4. I can identify instances where using the Health Improvement Card would improve patient outcomes
5. I can justify my reasoning for choosing to implement the Health Improvement Card with my patients
6. I understand when using the Health Improvement Card may **not** be appropriate for a particular patient
7. I can interpret the results and/or progress a patient using the Health Improvement Card in an accurate manner

Other comments:__________________________________________________________
