# Supplementary material for: Use of the Health Improvement Card by Chinese physical therapy students: A pilot study
Source: PLoS One. 2019 Sep 5;14(9):e0221630. doi: 10.1371/journal.pone.0221630 (PMC6728073; doi:10.1371/journal.pone.0221630)
Supplement: S4 Appendix — (PDF) [file pone.0221630.s004.pdf]

Raw data - students

Students	Sex	Age group	Height (m)	Weight (kg)	Waist Circumference (cm)	BMI	BMI risk level	Blood pressure risk level	Action duration 1	Diet risk level	Exercise risk level	Smoking risk level	Alcohol risk level	Action duration 2
S1	1	1	1.76	77	92.5	24.8	1	1	1	2	2	1	1	1
S9	1	1	1.76	72	79	23.24	1	1	1	2	1	1	1	1
S10	1	1	1.72	64	77.5	21.63	1	2	3	2	1	1	1	3
S11	1	1	1.7	66.5	81	23.01	1	1	1	2	1	1	1	1
S12	1	1	1.68	53	65	18.8	1	2	1	2	2	1	1	1
S21	1	1	1.65	52	71	19.1	1	1	1	2	1	1	1	1
S22	1	1	1.79	105	106	32.78	3	2	1	1	2	3	1	1
S23	1	1	1.7	63	80	21.7	1	1	1	2	1	1	1	1
S24	1	1	1.7	60	80	20.76	1	1	1	2	1	1	1	1
S25	1	1	1.62	55	71	20.96	1	1	1	2	2	1	1	1
S26	1	1	1.73	79	85	26.39	2	1	1	2	2	1	1	1
S27	1	1	1.72	65	82	21.97	1	1	6	2	2	1	1	1
T20	1	1	1.8	98	100	30.2	3	3	4	2	2	1	1	4
T21	1	1	1.63	80	100	30.11	3	3	1	2	1	1	1	1
T22	1	1	1.65	65	74	23.8	1	1	1	2	1	1	1	1
T23	1	1	1.72	60	77.5	20.3	1	1	3	2	1	1	1	3
T24	1	1	1.61	63		24.3	1	1	2	2	2	1	1	2
T25	1	1	1.77	70	84	22.3	1	1	3	2	1	1	1	1
T27	1	1	1.78	80	86	25.25	2	1	1	3	1	3	1	1
T28	1	1	1.75	80	90	26.12	2	1	24	2	1	1	1	24
T42	1	1	1.65	56	76	20.6	1	1	3	2	1	1	1	3
T46	1	1	1.8	76	78	23.4	1	1	1	2	1	1	1	1
T48	1	1	1.8	110	83.8	34	3	3	1	2	2	1	1	1
T50	1	1	1.8	70	75	21.6	1	1	1	2	1	1	1	1
T51	1	1	1.7	60	76	20.8	1	1	2	2	1	1	1	2
S2	2	1	1.58	54	75	21.6	1	1	1	2	2	1	1	3
S3	2	1	1.6	51	70	19.9	1	1	2	2	3	1	1	2
S4	2	1	1.65	47	67	17.3	1	1	1	2	3	1	1	1

S5	2	1	1.6	55	69	21.5	1	1	1	2	1	1	1	1
S6	2	1	1.65	55	72	20.2	1	1	1	2	3	1	1	1
S7	2	1	1.65	58	71	21.3	1	1	1	2	2	1	1	1
S8	2	1	1.56	47	65.4	19	1	1	1	2	3	1	1	1
S13	2	1	1.62	52	68	19.8	1	1	1	2	2	1	1	1
S14	2	1	1.58	55	75	22.03	1	1	6	2	3	1	1	6
S15	2	1	1.68	60	77	21.3	1	1	1	2	2	1	1	1
S16	2	1	1.73	85	95	28.4	1	1	1	2	2	1	1	1
S17	2	1	1.66	60	80	21.8	1	1	1	2	2	1	1	3
S18	2	1	1.58	50	64	20	1	1	1	2	3	1	1	1
S19	2	1	1.66	55	68	19.9	1	1	1	1	3	1	1	1
S20	2	1	1.6	55	68	21.5	1	1	1	2	2	1	1	1
S28	2	1	1.63	50	62	18.82	1	1	1	2	2	1	1	1
T1	2	1	1.64	53	69	19.7	1	1	4	2	2	1	1	4
T2	2	1	1.67	53	67	19	1	1	1	2	1	1	1	1
T3	2	1	1.73	66	70	22	1	1	1	2	1	1	1	1
T4	2	1	1.6	53	68	20	1	1	1	2	2	1	1	1
T5	2	1	1.67	54	67	19.4	1	1	1	2	2	1	1	1
T6	2	1	1.6	54	71	21	1	1	1	2	1	1	1	1
T7	2	1	1.56	45	65	18	1	1	1	2	2	1	1	1
T8	2	1	1.63	48	64	19.1	1	1	4	2	2	1	1	4
T9	2	1	1.7	64	72	22.14	1	1	4	2	1	1	1	4
T11	2	1	1.72	60	70	20.28	1	1	1	2	2	1	1	1
T12	2	1	1.6	41.1	64	16.2	1	1	4	2	1	1	1	4
T13	2	1	1.56	47.76	75	19.6	1	1	4	2	2	1	1	4
T14	2	1	1.58	48.5	66	19.5	1	1	4	2	2	1	1	5
T15	2	1	1.64	51	68	18.96	1	1	1	2	1	1	1	1
T16	2	1	1.7	55	72	19	1	1	1	2	1	1	1	1
T17	2	1	1.54	53	74	22.3	1	1	2	2	2	1	1	1
T18	2	1	1.59	54	75	21.36	1	1	1	2	3	1	1	1
T19	2	1	1.6	68	80	26.5	2	1	2	2	2	1	1	2
T26	2	1	1.73	55	65	18.38	1	1	2	2	2	1	1	2

T29	2	1	1.67	45	61	17.1	1	1	1	2	2	1	1	1
T30	2	1	1.72	74	93	25	2	1	3	2	1	1	1	5
T31	2	1	1.6	58	74	22.6	1	1	1	2	3	1	1	1
T32	2	1	1.6	57	77	22.3	1	1	1	2	3	1	1	1
T33	2	1	1.55	43.5	63	18.1	1	2	3	2	3	1	1	1
T34	2	1	1.62	47	62	18	1	1	1	2	1	1	1	1
T35	2	1	1.67	53	60	19	1	1	1	2	1	1	1	1
T36	2	1	1.72	56	76	18.92	1	1	1	2	2	1	1	1
T37	2	1	1.52	57	80	24.6	1	1	2	2	1	1	1	2
T38	2	1	1.7	55	65	19.03	1	1	2	2	2	1	1	1
T39	2	1	1.55	49	67	20.3	1	1	1	1	2	1	1	1
T40	2	1	1.63	56	68	21	1	1	1	2	1	1	1	1
T41	2	1	1.58	55	63	22.03	1	1	1	1	2	1	1	1
T43	2	1	1.66	48	68	17.4	1	1	1	1	1	1	1	1
T44	2	1	1.69	64	76	22.4	1	1	1	2	3	1	1	1
T45	2	1	1.63	78		29.36	2	1	3	2	2	1	1	1
T47	2	1	1.69	62	90	21.7	1	1	1	1	2	1	1	4
T49	2	1	1.65	52	74	19.1	1	1	1	1	1	1	1	1
T52	2	1	1.65	46	61	16.89	1	1	1	2	2	1	1	1
T53	2	1	1.59	49	65	19.8	1	1	1	2	1	1	1	2
T54	2	1	1.63	40	55	15.05	1	1	1	2	1	1	1	1
T55	2	1	1.59	49	65	19.8	1	1	1	1	1	1	1	2

Sex - 1=male; 2=female
Age group 1=20-34; 2=35-39; 3=40-44;4=50-54;5=55-59;6=60-64;7=65-69;8=70-74
BMI= body mass index
Risk level - 1=green code; 2=yellow code; 3=red code
Action duration 1=time committed to achieve BMI and BP to within low-risk zone
Action duration 2=time committed to achieve life-style status to within low-risk zone
