# Supplementary material for: Use of the Health Improvement Card by Chinese physical therapy students: A pilot study
Source: PLoS One. 2019 Sep 5;14(9):e0221630. doi: 10.1371/journal.pone.0221630 (PMC6728073; doi:10.1371/journal.pone.0221630)
Supplement: S5 Appendix — (PDF) [file pone.0221630.s005.pdf]

Raw data - friend / relatives

Friends /relatives	Sex	Age group	Height (m)	Weight (kg)	Waist Circumference (cm)	BMI	BMI risk level	Blood pressure risk level	Action duration 1	Diet risk	Exercise risk level	Smoking risk level	Alcohol risk level	Action duration 2
S1F1	2	1	1.68	55	80	19.49	1	1	1	2	3	1	1	9
S3F3	2	1	1.68	48	63.3	17	1	1	9	2	3	1	1	9
S5F5	2	1	1.61	48	70	18.52	1	1	1	2	1	1	1	1
S7F7	2	1	1.63	66.7	84	25.1	1	1	1	1	1	1	1	1
S9F9	2	2	1.63	53	70.2	19.9	1	1	1	2	3	1	1	1
S10F10	2	1	1.58	49	76	19.6	1	1	1	2	1	1	1	1
S13F13	2	4	1.6	55	70	21.8	1	1	1	2	1	1	1	1
S14F14	2	4	1.56	66	89	27.12	2	2	1	2	3	1	1	1
S15F15	2	1	1.58	50	74	20	1	1	1	2	3	1	1	1
S16F16	2	1	1.68	55	77	19.4	1	1	1	2	1	1	1	1
S17F17	2	4	1.6	63.5		24.8	1	2	6	1	2	1	1	1
S18F18	2	1	1.59	80	80	31.64	3	3	1	2	1	1	1	1
S19F19	2	1	1.72	66	73	21.23	1	1	1	1	2	1	1	1
S21F21	2	1	1.62	51	73	19.43	1	1	1	2	2	1	1	1
S22F22	2	1	1.55	47	56	19.6	1	1	1	1	1	1	1	1
S23F23	2	1	1.5	50	65	20	1	1	1	2	1	1	1	1
S24F24	2	1	1.6	44.5	71	17	1	1	1	2	3	1	1	1
S25F25	2	1	1.56	48	68	19.72	1	1	1	2	3	1	1	12
S26F26	2	1	1.65	60	65	22.04	1	1	1	2	1	1	1	1
S27F27	2	1	1.67	60	75	21.5	1	1	2	2	2	1	1	2
T2F2	2	1	1.6	50	70	19	1	1	1	2	1	1	1	1
T3F3	2	1	1.68	54	68	19	1	1	1	2	1	1	1	1
T5F5	2	1	1.6	51	67	19.9	1	1	13	2	2	3	1	13
T6F6	2	1	1.59	45	65	18	1	1	1	2	2	1	1	1
T7F7	2	1	1.5	46	65	20	1	1	7	1	1	1	1	7
T8F8	2	1	1.7	62	75	21.5	1	1	1	2	2	1	1	1
T9F9	2	1	1.65	50	70	18.4	1	1	1	2	1	1	1	1
T11F11	2	1	1.64	50	64	18.59	1	1	1	2	3	1	1	1

T13F13	2	1	1.56	47.76	75	19.6	1	1	4	2	1	1	1	4
T18F18	2	1	1.55	44	62	18.31	1	1	1	2	3	1	1	2
T21F21	2	4	1.65	55	68	20.2	1	1	1	1	1	1	1	1
T25F25	2	1	1.64	70	82	26	2	2	6	1	3	1	1	1
T29F29	2	1	1.71	74	72	21	1	1	1	2	2	1	1	1
T30F30	2	1	1.7	55.2	63	19.1	1	1	6	2	2	1	1	6
T33F33	2	1	1.66	52	70	20.3	1	2	1	2	2	1	2	1
T34F34	2	1	1.63	48	63	18	1	1	1	2	2	1	1	1
T35F35	2	1	1.64	51	68	18.96	1	1	1	2	1	1	1	1
T37F37	2	1	1.69	74	90	25.9	2	1	5	2	2	1	1	5
T38F38	2	3	1.57	59	69	23.6	1	1	1	2	1	1	1	life
T39F39	2	1	1.61	50	67	19.29	1	1	1	1	2	1	1	1
T40F40	2	1	1.64	51	68	18.96	1	1	1	2	1	1	1	1
T42F42	2	3	1.63	72	84	27.1	2	1	2	2	3	1	1	2
T43F43	2	1	1.68	55	69	19.5	1	1	4	1	1	1	1	3
T44F44	2	1	1.73	55	66	18.4	1	1	1	2	3	3	1	1
T45F45	2	1	1.63	78		29.36	2	1	1	2	3	2	1	1
T47F47	2	1	1.6	62	95	24.7	2	2	4	2	2	1	2	5
T48F48	2	1	1.6	45	40	18	1	1	1	2	2	1	1	1
T50F50	2	1	1.64	51	68	18.96	1	1	1	2	1	1	1	1
T52F52	2	1	1.7	60	70	20.76	1	1	4	2	1	1	1	4
T53F53	2	1	1.51	50	68	21.92	1	2	2	2	2	1	1	2
T54F54	2	2	1.61	80	70	23.14	1	2	4	1	1	1	1	4
T55F55	2	1	1.51	50	68	21.92	1	2	2	2	2	1	1	2
S2F2	1	1	1.72	62		20.9	1	1	3	2	2	2	1	1
S4F4	1	1	1.78	70.5		22.3	1	1	1	2	1	1	1	1
S6F6	1	1	1.76	52	65.2	17.37	1	1	1	2	2	1	1	1
S8F8	1	1	1.83	73	75	21.8	1	1	1	2	1	1	1	1
S11F11	1	1	1.78	73	86	23.04	1	2	1	2	2	1	1	1
S12F12	1	1	1.72	66	76	22.3	1	1	1	2	1	1	1	1
S20F20	1	1	1.73	65	75	21.7	1	1	1	2	1	1	1	1
T4F4	1	4	1.75	80	101	26	2	2	2	2	2	1	2	2

T10F10	1	1	1.75	59		19.27	1	1	2	2	3	1	1	2
T12F12	1	1	1.75	63	75	21	1	1	4	2	3	3	2	4
T14F14	1	1	1.71	58	72	19.8	1	1	2	1	1	2	1	2
T15F15	1	1	1.83	70	75	21.6	1	3	1	2	1	1	1	1
T16F16	1	1	1.75	65	81	21.2	1	1	2	2	2	1	1	2
T17F17	1	1	1.7	88	97	30.6	3	1	1	2	2	1	1	1
T19F19	1	1	1.73	70	79	23.38	1	1	12	3	3	3	3	12
T20F20	1	1	1.8	60	75	18.6	1	1	1	1	1	1	1	1
T22F22	1	1	1.75	60	72	19.5	1	1	1	2	1	1	1	1
T23F23	1	1	1.82	100	105	30.1896	3	1	6	2	1	1	1	1
T24F24	1	1	1.81	90	80	27.5	1	1	2	1	1	1	1	2
T26F26	1	1	1.78	82	92	25.9	2	2	2	2	3	1	1	1
T27F27	1	3	1.73	79	86	26.39	2	1	1	2	1	3	2	1
T28F28	1	1	1.78	70	70	22.09	1	1	5	1	1	1	1	5
T31F31	1	1	1.72	75	85	25.35	2	2	life	2	2	1	1	life
T32F32	1	3	1.72	70	70	23.2	1	1	1	2	3	3	1	1
T36F36	1	1	1.78	70	90	22	1	2	3	3	1	1	1	1
T41F41	1	2	1.78	80	93.9	25.24	2	2	5	2	3	2	2	5
T46F46	1	2	1.66	42	70	25.3	1	1	1	1	1	1	1	1
T49F49	1	1	1.7	58	78	20	1	1	1	2	3	3	3	1
T51F51	1	1	1.74	71	72	13.45	1	2	1	1	2	1	1	1

Sex - 1=male; 2=female

Age group 1=20-34; 2=35-39; 3=40-44;4=50-54;5=55-59;6=60-64;7=65-69;8=70-74

BMI= body mass index

Risk level - 1=green code; 2=yellow code; 3=red code

Action duration 1=time committed to achieve BMI and BP to within low-risk zone

Action duration 2=time committed to achieve life-style status to within low-risk zone
