# Supplementary material for: Use of the Health Improvement Card by Chinese physical therapy students: A pilot study
Source: PLoS One. 2019 Sep 5;14(9):e0221630. doi: 10.1371/journal.pone.0221630 (PMC6728073; doi:10.1371/journal.pone.0221630)
Supplement: S6 Appendix — (PDF) [file pone.0221630.s006.pdf]

	Q1	Q2	Q3	Q4	Q5	Q6	Q7
S1Q1	A	A	A	A	A	A	DA
S2Q1	SA	SA	SA	A	A	DA	DA
S3Q1	SA	SA	SA	SA	SA	A	A
S4Q1	SA	SA	SA	A	SA	A	A
S5Q1	SA	SA	SA	A	SA	SA	A
S6Q1	SA	SA	SA	SA	SA	SA	DA
S7Q1	SA	SA	SA	A	SA	SA	A
S8Q1	SA	SA	A	A	SA	A	A
S9Q1	SA	SA	A	A	A	SA	A
S10Q1	SA	SA	A	A	A	A	A
S11Q1	SA	SA	SA	SA	A	A	A
S12Q1	SA	SA	SA	SA	SA	A	A
S13Q1	A	A	DA	A	A	A	DA
S14Q1	A	DA	A	A	DA	A	DA
S15Q1	SA	SA	SA	SA	SA	A	SA
S16Q1	SA	SA	SA	A	SA	A	SA
S17Q1	SA	SA	SA	SA	SA	SA	A
S18Q1	SA	SA	SA	SA	SA	A	A
S19Q1	SA	SA	SA	A	SA	SA	A
S20Q1	SA	SA	A	A	A	A	DA
S21Q1	SA	A	A	A		A	A
S22Q1	A	SA	SA	SA	SA	SA	DA
S23Q1	SA	SA	A	A	SA	A	DA
S25Q1	SA	SA	SA	A	SA	A	A
S26Q1	SA	SA	SA	A	A	A	A
S27Q1	SA	SA	SA	SA	SA	SA	SA
T1Q1	SA	SA	SA	SA	SA	SA	SA
T2Q1	SA	SA	SA	SA	SA	SA	SA
T3Q1	SA	SA	SA	SA	SA	SA	SA
T4Q1	SA	DA	SA	DA	SA	DA	DA
T5Q1	SA	DA	DA	DA	SD	A	DA
T6Q1	A	A	A	A	A	SA	A
T7Q1	A	A	A	A	A	A	A
T8Q1	SA	A	A	A	A	A	A
T9Q1	SA	SA	SA	SA	SA	SA	SA
T10Q1	SA	A	A	A	A	A	A
T11Q1	SA	A	A	A	A	A	A
T12Q1	SA	A	A	A	DA	DA	DA
T13Q1	SA	DA	A	A	A	A	DA
T14Q1	A	DA	DA	A	DA	DA	DA
T15Q1	SA	SA	SA	A	A	A	
T16Q1	SA	SA	SA	A	SA	A	A
T17Q1	SA	A	A	A	SA	A	A
T18Q1	SA	A	SA	SA	SA	SA	A
T19Q1	SA	A	SA	A	SA	A	A
T20Q1	A	A	A	A	A	A	A
T21Q1	SA	A	A	DA	A	DA	DA

T22Q1	A	A	A	A	A	A	A
T23Q1	SA	SA	A	A	DA	A	A
T24Q1	SA	SA	SA	A	SA	SA	SA
T25Q1	SA	A	A	A	SA	A	A
T26Q1	SA	A	SA	A	SA	SA	SA
T27Q1	SA	SA	SA	SA	A	SA	A
T28Q1	SA	SA	SA	SA	SA	SA	SA
T29Q1	A	DA	A	DA	DA	SD	SD
T30Q1	A	DA	A	A	A	DA	DA
T31Q1	SA	SA	SA	SA	SA	SA	SA
T32Q1	SA	SA	SA	SA	SA	A	SA
T33Q1	A	A	SA	A	SA	A	DA
T34Q1	SA	A	SA	SA	SA	SA	A
T35Q1	SA	SA	SA	SA	SA	SA	A
T36Q1	SA	A	A	A	A	A	A
T37Q1	SA	SA	SA	SA	SA	A	A
T38Q1	SA	SA	SA	SA	SA	SA	A
T39Q1	A	A	A	A	A	DA	A
T40Q1	SA	SA	SA	A	SA	A	A
T41Q1	SA	SA	SA	SA	SA	A	SA
T42Q1	SA	SA	A	A	SA	SA	A
T43Q1	SA	A	A	A	SA	A	A
T44Q1	A	A	A	A	SA	SA	A
T45Q1	SA	A	SA	A	A	A	A
T46Q1	A	A	A	A	A	A	A
T47Q1	SA	A	A	SA	A	A	A
T48Q1	SA	SA	SA	SA	SA	SA	SA
T49Q1	SA	A	SA	SA	A	DA	DA
T50Q1	A	A	A	A	A	A	A
T51Q1	A	A	A	A	A	A	A
T52Q1	SA	SA	SA	SA	SA	SA	SA
T53Q1	SA	A	A	A	A	A	A
T54Q1	SA	SA	SA	SA	SA	SA	SA
T55Q1	SA	SA	A	A	A	SA	DA
counts							
SA	64	44	44	28	44	28	16
A	17	30	34	49	30	44	45
DA	0	7	3	4	5	8	18
SD	0	0	0	0	1	1	1

SA=strongly agree; A=agree;D=disagree;SD=strongly disagree

Q1=Physiotherapists should introduce the Health Improvement Card to the general public

Q2=I understand the purpose and role of the Health Improvement Card

Q3=I can provide advice to my patients about the actions prescribed on the Health Improvement Card

Q4=I can identify instances where using the Health Improvement Card would improve patient outcomes

Q5=I can justify my reasoning for choosing to implement the Health Improvement Card with my patients

Q6=I understand when using the Health Improvement Card may not be appropriate for a particular patient

Q7=I can interpret the results and/or progress a patient using the Health Improvement Card in an accurate manner
