# Supplementary material for: Use of the Health Improvement Card by Chinese physical therapy students: A pilot study
Source: PLoS One. 2019 Sep 5;14(9):e0221630. doi: 10.1371/journal.pone.0221630 (PMC6728073; doi:10.1371/journal.pone.0221630)
Supplement: Attachment — Submitted filename: Responses to Reviewers.docx [file pone.0221630.s007.docx]

**Responses to Editor’s comments:**

1. To obtain a DOI from protocols.io so that our protocol can be sited independently in the future.

***Good suggestion. We have now obtained a DOI for our protocol. This information is included in the unmarked manuscript page 4, lines 63-64, under Materials and methods.***

2. Please ensure that your manuscript meets PLOS ONE's style requirements, including those for file naming.

***The format of our manuscript follows the two sample files as suggested. We have included a statement acknowledging the funding resource on the Title page and inserted the symbol “¶” to indicate equal contribution of our authors (page 1 of the unmarked manuscript)****.*

3. Please update the ethics statement on the submission form and the methods section of your manuscript file to remove the word "Blinded" and replace with the named IRB that approved your study.

***Edited (unmarked manuscript page 4, under Materials and methods, Lines 61-63.***

**Responses to Reviewers’ Comments**

**Reviewer 1:**

We are encouraged that the merit of work related to better educating physical therapy students about their role in addressing NCD risk, is shared.

a) Please indicate n= in the text of Results - currently only percentages are presented.

***The numbers of responses from students have been included (page 6, lines 115-116; page 7, lines 138 & 139; page 8, lines 156-159).***

b) Table 1 - there are no data presented for males under the column labelled 'Action duration 2' - please revise and add.

***Edited (Table 1, page 7).***

c) The use of abbreviations introduced into the text is not consistent throughout the different sections of the manuscript - please revise.

***Reviewed and edited.***

d) Grammatical errors:
Line 205 - should be 'professionals'

***Edited (page 12, line 214).***

Line 222 - should be 'therapists'

***Edited (page 12, line 230).***

Lines 230 - 236 - please revise sentence construction and language use

***The wordings in this paragraph has been edited (page 13, lines 247-254).***

e) Conclusion: consider re-writing in a more concise manner.

***The Conclusion has been rewritten to be more concise (page 14-15, lines 274-288).***

**Reviewer 2:**

The title indicates this is a pilot study; as such the feasibility and next steps in moving to a bigger study are not fully considered in the discussion (there is a line about on time/burden which is helpful).

***Based on the positive findings of this pilot study, we have now commenced our work in a community project, with physical therapy students involved in provision of health education and exercise advice to members of the general public attending a community centre. We have better elaborated on this and the implications of the findings in the Discussion (page 13-14, lines 260-267).***

Conclusion in the Abstract (Line 22) and the main conclusion (line 257) – it is not quite clear what the term re-evaluation means. Is it responsiveness i.e. whether the HIC is responsive to change following an intervention?

***We have edited this sentence and replaced ‘re-evaluation’ with ‘tracking the progress over time’.***

Introduction
line 46 ‘receptive’ vs 'receptive to' or 'able to'

***Edited (page 4, line 52).***

Lines 51-54 appear to include the study aims but these seem a bit different to the start of the abstract; consistency and explicit identification of the study aims would be helpful.

***The first 3 lines of the Abstract have been edited. The aim of the study in the Abstract and Introduction is now consistent.***

Methods
Line 75 “height and weight were self-reported” Perhaps this should be considered/discussed as a potential limitation to the study, given that frequently there are inconsistencies between self-report and accurate measurement. Physiotherapy students should be able to measure height and weight with minimal equipment/expense but perhaps I am assuming this for all undergraduate programmes.

***We have now included this as a limitation of the study in the Discussion (page 13, lines 255-259).***

Line 85 “instruction provided identify” Vs “instruction provided to identify”

***Edited (page 5, line 88).***

Results
Line 124 “she” vs “they were”

***Edited to ‘this student’ (page 7, line 134).***

Discussion
Line 191 “….reverse the tide of ….” Please consider use of more scientific language here

***Edited (page 11, lines 205-206).***

Line 209 “given” Vs “as”

***Edited (page 12, line 224).***

224-225 – This is not quick clear. What does “order” mean; does it mean to request these results from a medical site?

***Edited (page 13, lines 240-241).***

Line 230 “That the students….” Rephrase the start of this sentence please

***Edited (page 13, lines 247-248).***

233 – Does this mean the authors will now contact the WHPA with these results, it may be worth considering whether a recommendation can be made at this stage based on a pilot study.

***The student’s reflections are being forwarded to the WHPA for their consideration in the next iteration of the Card. We consider their suggestions highly relevant and will enhance its utility overall (page 13, lines 244-246).***
